# Disability: a welfarist approach

**DOI:** 10.1258/ce.2011.011010

**Published:** 2011-03

**Authors:** Julian Savulescu, Guy Kahane

**Affiliations:** Oxford Uehiro Centre for Practical Ethics, University of Oxford, Littlegate House, St Ebbes Street, Oxford OX1 1PT, UK

## Abstract

In this paper, we offer a new account of disability. According to our account, some state of a person's biology or psychology is a disability if that state makes it more likely that a person's life will get worse, in terms of his or her own wellbeing, in a given set of social and environmental circumstances. Unlike the medical model of disability, our welfarist approach does not tie disability to deviation from normal species’ functioning, nor does it understand disability in essentialist terms. Like the social model of disability, the welfarist approach sees disability as a harmful state that results from the interaction between a person's biology and psychology and his or her surrounding environment. However, unlike the social model, it denies that the harm associated with disability is entirely due to social prejudice or injustice. In this paper, we outline and clarify the welfarist approach, answer common objections and illustrate its usefulness in addressing a range of difficult ethical questions involving disability.

Many people believe that it would be wrong deliberately to create a child with deafness or dwarfism when it is possible to create a healthy, hearing child.^1^ They believe that such procreative choices are wrong because deafness and dwarfism are disabilities – conditions that are abnormal and negatively deviate from normal human species’ functioning and which are therefore harmful and which we should generally try to prevent or correct. This set of beliefs is formalized as the ‘medical model’ of disability. By contrast, those who want to have children with these conditions vehemently deny that there is anything inherently undesirable about deafness or dwarfism. They often appeal to what some disability advocates call the ‘social model’ of disability, according to which deviation from normal human functioning is disadvantageous – if disadvantageous at all – *only* because of social prejudice. These are two sides to a longstanding and bitter dispute about the concept of disability.^2^

Our aim in this paper is not to criticize existing approaches but to propose an alternative approach: the welfarist account of disability.^3^ The advantage of the welfarist approach, we believe, is that it incorporates the insights contained in both the medical and social models but avoids their more implausible aspects.^4^ Like the social model, the welfarist approach denies that normal functioning is in itself morally significant.^5^ But like the medical model, it does not take social prejudice to be the sole or even key source of the disadvantage associated with disability.

In what follows, we introduce the welfarist approach and explain its main features. We believe that common conceptions of disability, encapsulated by the medical model, are often an obstacle to clear ethical thinking.^6^ This is why, like the social model, the welfarist approach is revisionist: it is not meant as a straightforward analysis of the everyday use of the word ‘disability’. We end this paper by illustrating how our approach sheds better light on controversial reproductive choices and some other ethical disputes about disability – and thus, we hope, also demonstrate why the current understanding of disability should be revised.

## Introducing the welfarist account

According to the welfarist account of disability, ‘disability’ should refer to any stable physical or psychological property of subject S that leads to a significant reduction of S's level of wellbeing in circumstances C, *excluding* the effect that this condition has on wellbeing that is due to prejudice against S by members of S's society.^7^ Several aspects of this account require clarification.

### Nothing to do with normality

A ‘stable physical or psychological property’ of a person is not the same as an ‘impairment’, if this notion is taken to be synonymous with some deviation from normality. We are referring to any intrinsic property of the agent. Thus, unlike the traditional medical model, the welfarist account makes no reference to biological or statistical normality: whether or not a condition is normal or deviates from normality is *not* an intrinsic property of a person. This means that, as we shall see, some things will count as disabilities in our sense that we might not describe as disabilities in the everyday sense of the word.

### In-built normativity

Although our definition does not use explicit evaluative and normative terms, we do refer to the concept of wellbeing, which has inherent normative significance. If something leads to a reduction in someone's wellbeing, then that thing is bad for that person. This means that if something is a disability in our sense, then it is also by definition a condition that makes life worse, and gives *prima facie* reasons to address it. In this respect, the welfarist approach captures the thinking behind clinical specialties at the fringes of conventional medicine, especially palliative medicine, in which a patient's own conception of wellbeing is taken by clinicians to be normative, and treatment offered only if it will improve it.

### Intrinsic value

Our definition refers to what is of intrinsic value: wellbeing. When something reduces someone's wellbeing, then what is intrinsically bad is the harm it does – the reduction of wellbeing. Importantly, the *cause* of that reduction is only *instrumentally* bad or harmful. While we may have some reason to correct it *as a means* to removing the intrinsic harm, we could equally remove the harm by changing the *circumstances* in which the effects are rendered harmful. There is thus no overriding reason to correct conditions that count as disabilities in the welfarist sense. The question whether to change a person's biology psychology or environment will be determined by considerations such as cost, safety, ease and justice.^8^

### Context dependence

According to the welfarist account, disability in our sense is relative to both persons and circumstances (we refer to circumstances C, but this could stand for a long list). In its everyday use, ‘disability’ is not context-dependent. Everyday use not only mistakenly implies that deviations from the species norm are bad, but also seems to imply that they are bad in themselves, quite independently of context.

On the welfarist account, by contrast, disability is context-dependent. What makes leading a good life harder in one circumstance, may make it easier in another. The disposition to rest and store excess calories as fat is an advantage in a world of limited resources but a serious disadvantage in a world of excess calories and limited physical activity. Deafness would be an advantage in an environment of extremely loud and distracting noise.^9^ In order to judge which conditions constitute a disability, we need to know what class of people is being referred to, and to predict what the context or environment is likely to be. There is no context-independent answer to such a question.

We deliberately defined disability to be relative to a specific person and set of circumstances. When we consider questions about individuals’ own wellbeing, this is the normative reference point – the wellbeing of the ‘average’ or ‘common’ person has no independent moral standing. Indeed, it has no direct relevance. That there are many people starving does not make my starvation less bad for me. Nevertheless, for the purposes of social policy, we often need to speak in generalities. Certain foods, substances, activities, temperatures, etc. are harmful to *most* human beings. But that does not change the point that many things can be harmful or disabling to one person but not to another; harmful to a person at one time but not in another; or harmful in one set of circumstances and not in another. Folate is generally beneficial to people, important for health and the prevention of birth defects, but if given to a person with vitamin B_12_ deficiency, it can be lethal.

### Excludes social prejudice

We have deliberately defined ‘disability’ in a way that excludes the effect on wellbeing that a condition has as a result of social prejudice. Advocates of the social model are no doubt right that some of the adverse effects of deviation from the species norm are due to such prejudice. We disagree, however, that *all* of the ways in which common disabilities can reduce wellbeing are *entirely* due to prejudice.^10^ And although our welfarist account is somewhat revisionary, to define ‘disability’ to refer exclusively to such prejudice seems *too* revisionary. Talk about prejudice towards disabled people would be a tautology, and it would thus make no sense to say that some society is prejudiced against disabled people. Moreover, although our account makes it clear that disabilities reduce wellbeing only in a given context, we deny that this context must always be *social*, or that when it is social, it must always reflect *prejudice* or *injustice*. Even if many of the limitations imposed by deafness are due to the fact that many social arrangements are designed for hearing people, it does not automatically follow that all of these social arrangements are unjust.^11^

#### Everyone suffers from disability

An atopic constitution (the tendency to develop allergic responses) is a disability in modern society. It can result in asthma and eczema. Asthma is instrumentally bad because it makes breathing more difficult in certain environments commonly encountered in the developed world – dusty or smoky environments or places with pets. It can make it more difficult to enjoy the company of others or do physical activity if it is difficult to breathe. About 20% of people who have asthma nowadays are mildly disabled by virtue of it. Myopia is an example of another common but very mild disability. Dyslexia is another. *All of us* can be said to suffer from disabilities – conditions inherent to our nature which reduce our wellbeing and make it more difficult to realize a good life in the context we inhabit.

On the welfarist account, illness is a form of disability. The priority which we attach to disease or illness being treated or researched is proportional to the degree to which it is disabling. Some illnesses have so little impact on a person's life that such ill people have very little claim to treatment, despite the fact that their biological functioning might significantly deviate from the species-typical norm.^12^

People sometimes associate disability with visible and overt features of people's bodies, or with very severe mental limitations. But genetics, biology and psychology will identify many other internal features of people to be impediments to wellbeing. It may turn out, for example, that having poor impulse control is, in many contexts, a far greater obstacle to a good life than being deaf or missing an arm.^13^ That is, having poor impulse control may adversely affect wellbeing, and thus be a *far greater* disability than losing an arm, even if the intuitions of many will militate against this claim. The fact that certain properties of people are more salient than others may distort our understanding of the weight they have in shaping people's lives and the proper prioritization of medical research and treatment. Our welfarist account tries to correct this distortion by defining disability in a broader and more inclusive way.

More importantly, it provides a metric for prioritizing medical research and treatment, along the lines of the ‘global burden of disease’ but with a firm and appropriate focus on what ultimately and intrinsically matters: human wellbeing.

### Reference to wellbeing

To determine whether some condition counts as a disability in the welfarist sense, we need to conduct two separate inquiries, one normative and one empirical. First, we need to agree upon and adopt an account of wellbeing. Then we need to identify the causal factors that influence a person's wellbeing in a certain set of circumstances.

It is thus a substantive question, not determined by definition alone, whether the paradigmatic cases of disability in the everyday sense – e.g. deafness, blindness and cognitive impairment – are disabilities. We ourselves are inclined to believe most of these are, in the conditions holding at present and in the foreseeable future. But note that although it might be possible to make a general case that, say, blindness is a disability for most people in the common circumstances holding in our world, it may still be true that an opposite case can be made for a *particular* person, in special and specific circumstances. For example, it may be true that for a person living exclusively and sufficiently within a well resourced deaf community, deafness might be a trivial disability.^14^

## Objections to the welfarist account

### Does it prejudge the normative issues?

Since on our definition it is tautological that disability is bad for those suffering from it, and we have reasons to remove disabilities, this account might be thought to foreclose certain genuine normative questions about disability, about whether disabilities are genuinely bad or harmful. The normativity is already written into the concept.

However, we believe that the inherent normativity is a strength, not a weakness. Moore's Open Question Argument easily reveals that ‘disability’ is a normative concept in ordinary usage: it would make no sense to say, ‘I know that X is a disability, but is it bad?’ Importantly, our definition does not foreclose any genuine normative question. On the contrary, it makes it impossible to move from a certain neutral relational empirical property (deviation from the norm) to a tendentious normative conclusion simply through a semantic detour. But the conceptual tie between wellbeing, value and reasons is acknowledged by any sane normative view.

What our definition does is direct attention to the normative questions that really matter here: questions about what affects human wellbeing. This, we believe, is of central importance. But once empirical facts are understood and an account of wellbeing is agreed, there is no further intrinsic normative issue about disability that remains open.^15^ There are of course other normative considerations that might be relevant: other people's wellbeing, distributive justice, desert or other issues given importance by an individual's moral theory. But these other considerations were there anyway.

Two further remarks. First, as noted above, although disability implies reasons to change the situation – reasons to improve wellbeing – it does not necessarily imply reasons to correct the disabling *condition*. It leaves open, as it should, the question whether to change the person or his/her environment. Second, the reasons issued are in any case only *prima facie* reasons. The conceptual tie is not to what one has *conclusive* reason or what one necessarily *ought* to do. Thus, while there will always be *some* reason to avoid disability (by changing either the person or environment), there may sometimes be overall reasons to accept or sometimes even promote disability in a given context. The normative questions are not at all foreclosed at *this* level.

### Isn't it useless, because there is too much disagreement about what makes one person's life better than another?

What constitutes a good life is a difficult philosophical question.^16^ It has also been the subject of extensive sociological, anthropological, psychological and economic enquiry. One of the most urgent tasks, according to the welfarist account, is to agree upon a substantive account of what makes a human life go well. This is a deeply disputed but nonetheless urgent and ultimate question.

Within philosophy, over the last 2500 years, there have been three dominant approaches. According to hedonistic theories, it consists of having pleasant experiences and being happy. According to desire-fulfilment theories, what matters is having our preferences fulfilled. According to objective good theories, certain activities are intrinsically good – developing deep personal relationships and talents, gaining knowledge, and so on. But although these are opposing accounts of the nature of wellbeing, they might nevertheless often significantly agree about what *particular* things *make* life go better or worse. Thus, for example, hedonism and desire-satisfaction theories typically claim that significant relationships and achievements are good because they give us pleasure or satisfy our strong desires. Objective good theories typically recognize the value of pleasure and pain and think that our *informed* desires often track what is independently good. This convergence is not surprising, since these are all competing accounts of our everyday concept of wellbeing, and as such are likely to preserve many of our pre-theoretical beliefs about wellbeing – beliefs that are indeed diverse, but also share a large core. To fail to see this is to fail to distinguish the *theory* of wellbeing and the *sources* of wellbeing.

It is important to stress that while there is disagreement about wellbeing, there is also much consensus. Few if any would deny that chronic pain tends to make a life worse, or that joy makes a life better. All plausible moral theories have to make such judgements – judgements about harms and benefits, or things that make a life go better or worse. Our welfarist account does not rely on some special and controversial conception of wellbeing. All it asks us is to apply the same concepts we already employ in everyday situations. And every day we make implicit judgements about what is good and better for people when we form social policies, develop priorities for research, educate our children and prioritize social institutions and practices.

## The welfarist account in action

We will now briefly consider some examples of ethical disputes involving disability, and illustrate how they should be interpreted in light of the welfarist approach. We believe that although our account sometimes leads to surprising conclusions, it does a better job than the Medical and Social models, or than the loose everyday notion of disability, in highlighting what is of genuine ethical significance in each of these cases.

As we saw, to apply the welfarist account we need to give answers to questions about wellbeing. But few would deny that, in most cases, we can at least give rough answers to questions about wellbeing, and if this is the case, then we can also give rough answers to questions about disability. In this section we will examine a number of interesting cases and give such rough answers. But these are just rough answers. There are no general armchair answers to questions about disability in our sense.

### Deafness and dwarfism

Sharon Duchesneau and Candy McCullough, a deaf lesbian couple, had their second child Gauvin in 2001. The women, who wanted to have a deaf child, conceived Gauvin through artificial insemination by donor, using sperm from a friend they knew to have five generations of inherited deafness in his family. They argued that deafness is an identity, not a medical affliction that needs to be fixed. As they put it, ‘Deafness is not a disability’. A hearing child would be a blessing, they said, but a deaf child would be a special blessing.^17^

Some achondroplastic dwarfs have similarly sought preimplantation genetic diagnosis to select an embryo with dwarfism, arguing that being little is not a disability, but only a difference. They have claimed that as their house and lifestyle have been modified for their short stature, they would be better able to rear a short child rather than a normal child – that if their child had achondroplasia, it would have a better life.^18^

What are we to make of such procreative decisions? Deafness and dwarfism are obviously deviations from the species norm. But are they also disabilities in our sense?^19^ It is arguable that deafness is instrumentally harmful in two senses. First, it reduces the goodness of a life by preventing access to the world of sound. A deaf person cannot hear music, human voice or auditory alarms. When studying in a library, deafness is not a disability – it may even be advantageous. It is the exercise of capacity to hear that is valuable, not the capacity itself. But the capacity to hear is, obviously, a necessary condition for enjoying those intrinsic goods that are necessarily auditory. And in *the world the way it is*, there are plenty of such goods. Second, deafness also reduces the chances of realizing a good life because it makes it harder to live, to achieve one's goals and to engage with others in a world which is based on the spoken word. Being able to hear is not a necessary condition for such activities and goods. But without that ability it is nevertheless significantly harder to move in the world, harder to respond to emergencies where the alarm is aural and so on. These difficulties are partly due to social circumstances, but, as pointed out earlier, this need not mean that they are all due to injustice. It is also tendentious to claim that the failure of a deaf person to hear the roar of an approaching tiger is the result of social construction. Of course, the world could be constructed with visual warnings of approaching tigers, but the world could be constructed in a near infinite number of ways. Such an approach would make all harm socially constructed.

These general claims, however, are compatible with the claim that for particular people, in particular circumstances, deafness is *not* a genuine disability, as we have pointed out. Indeed, for adults whose life projects are closely tied with their condition, and who will need to make a difficult and painful transition to the world of hearing, remaining deaf might be preferable to becoming hearing. For these people, *hearing itself* would count as a kind of disability.

Similar considerations apply to dwarfism. To us this seems at most a mild disability, continuous with different limitations on wellbeing that all of us have (it makes no difference to wellbeing whether a person's short stature is due to a genetic abnormality or to normal genetic variance). We doubt that achondroplasia does much to reduce the quality of a person's life once we subtract the consequences of prejudice.^20^

### Body integrity identity disorder

Consider next an even more controversial case. There is a small minority of individuals who are sometimes described as ‘want-to-be amputees’. These individuals do not identify with the body they were born with: a body with four limbs. They want to remove one or more of their healthy limbs. For at least some of them, this desire persists even after extensive counselling and psychiatric therapy, and they find life with four limbs extremely depressing.^21^

In most cases, losing one's limb would be a significant disability, and it would consequently be a serious prudential mistake to try to amputate one's own limb, or even simply to risk losing it. But in the case of some of these would-be amputees, it might actually be a disability to *possess* a healthy limb, if indeed the psychological distress is severe enough and not correctable. In these different contexts, the same condition might amount in one case to a harm and in another to a benefit, and what would count as ‘correcting’ a disability would be very different. Once we drop the instinctive reliance on normality as a normative guide, this result should not be so surprising. It is not intrinsically bad to have only one leg just as it is not intrinsically bad to have ‘only’ two, or three and not eight.

Of course, to lose a leg is at least potentially to lose a degree of mobility, and consequently some degree of wellbeing. To what extent this is a loss will depend on the sophistication of the prosthetic legs available (most would-be amputees apparently have no qualms about using prosthetic limbs), and as technology advances, the negative effect on mobility will continue to diminish. Indeed, as technology matures, prosthetics at some point may surpass normal-functioning limbs. Then having a normal leg would be disability for most people. But even in the current case where prosthetic limbs are inferior in functioning to normal limbs the negative effect of having a prosthetic leg could still be less than the negative effects of *keeping* the leg.^22^

This is just a hypothesis. To properly assess it, we would need to engage in serious empirical legwork.^23^ It cannot be decided from the armchair, from sketchy case descriptions, let alone by gut reactions. We need to overcome gut responses to surgery or to the ‘deformation’ of the human figure, and to ask instead what effect such surgery would plausibly have on particular people's wellbeing.

### The ‘Ashley treatment’

In 2004, Ashley was a nine-year-old girl from Seattle who was born with static encephalopathy, a severe brain impairment that left her unable to walk, talk, eat, sit up or roll over. Ashley would remain at a developmental level of a three-month-old baby. In that year, Ashley was given high-dose oestrogen therapy to stunt her growth, and her uterus and breast buds were removed to prevent menstrual discomfort and to limit the growth of her breasts. Ashley's parents argued that this treatment was intended ‘to improve our daughter's quality of life and not to convenience her caregivers’.^24^

On both our welfarist account and on the medical model, Ashley was born with a severe disability.^25^ But their verdicts radically diverged when applied to the ‘Ashley treatment’. On the medical model, the treatment would greatly increase her disability – driving her even further from the human norm. In our view, in the *context* of Ashley's brain impairment, and assuming that the claims made for the effects of the treatment on Ashley's wellbeing were correct, the treatment would be not disabling but enhancing.

We think that the welfarist concept of disability does a better job, and sheds more light on the Ashley case, than the medical model. As for the social model, it has been claimed by some that Ashley's condition is detrimental only because of adverse social circumstances and that it is only these circumstances that need to be changed – for example by providing further support for the parents to lift or transfer Ashley, and so forth. This claim is implausible. Not all the detrimental aspects of Ashley's condition are due to lack of social support, nor should it be simply assumed that changes to the social circumstances are always to be preferred or just.^26^

### IQ reduction

Cardiac bypass is well known to have been associated with cognitive impairment. Imagine that a man, Jones, a man with heart disease, undergoes coronary artery bypass grafting for ischaemic heart disease. He is not told of the possibility of cognitive impairment and suffers some degree of impairment of memory, compared with previously. He sues, claiming that his doctors disabled him. IQ testing reveals that his IQ has dropped from 160 to 140.

Is it a disability to have an IQ of 140 instead of 160? The average IQ is 100 and an IQ of 140 is still in the top 2%. However, if a drop of 20 points of IQ reduces one's prospects for the best life, if it impedes one's progress, even slightly, if it makes it less likely one will achieve one's goals, then it reduces the expected value of one's life and constitutes the infliction of a disability, even if only minor. We concede that this will sound odd to many people. But being less intelligent might have a far greater impact on a person's life than having only three limbs. What kind of impact this might have on a person's wellbeing is largely an empirical question, and there is at least some evidence at hand that can help us answer it.^27^ Let us just point out two general considerations. First, intelligence is at least partly a positional good. The negative impact on a person's life of such a drop in intelligence would to a large extent depend on the intelligence levels of the people around him. In our world the negative impact might be minor. In a world where most people have 160 IQ, it would be very substantial. Second, it makes a difference at what point in one's life one suffers the drop in IQ. For the mature painter, becoming colour blind might be debilitating. For a professional mathematician, a significant drop in IQ is likely to be at least equally devastating. But it would not be equally harmful if it happens to a very young child.

## Conclusion

Discussion of disability has sometimes taken the form of an acrimonious and seemingly irresolvable debate between essentialists who think that deviation from a species norm or other standard of normality is intrinsically bad and always merits correction, and disability advocates and proponents of the social model who think that to be disabled is merely to be different, and that if there is harm associated with it this is always due to social prejudice. We believe that this is not a useful way to frame the debate. As we have argued, there is an element of truth in the social model. Conditions are disabling only in a given context. But essentialists are also partly right given that, in the circumstances obtaining in our world and in the likely future, it would be better if many commonly recognized disabilities were prevented or corrected.

Conceptions of disability that associate it with deviation from the normal are entrenched in public discourse, medicine and law. It would take dramatic and unlikely conceptual revolution for these to be replaced by our revised welfarist account. And proponents of the social model might argue that their conception of disability has played an important part in the fight for disability rights. Our aim in proposing a welfarist approach, however, is somewhat more modest. We have suggested that in a range of cases where difficult ethical questions arise in connection with disability, these questions are best approached by considering ways in which various conditions are likely to affect wellbeing. Reference to normal species functioning is, at best, redundant, and often obscures what is really at issue.
